# Giant cystic lymphangioma in the pancreatic tail–splenic hilum region managed by en bloc resection: A case report

**DOI:** 10.1097/MD.0000000000051000

**Published:** 2026-10-09

**Authors:** Yuting Wang, Bin Xiong, Junwei Niu, Shengwei Li

**Affiliations:** a Department of Hepatobiliary Surgery, Chongqing Medical University, Chongqing, China; b Department of Hepatobiliary Surgery, The Second Affiliated Hospital of Chongqing Medical University, Chongqing, China.

**Keywords:** cystic lymphangioma, pancreas, pancreatic tail, spleen, splenic hilum

## Abstract

**Rationale:**

Lymphangioma is a benign lymphatic malformation that occurs predominantly in children and at superficial sites. Intra-abdominal lymphangioma is uncommon, and involvement of the pancreatic tail–splenic hilum region is particularly rare. Such lesions may mimic pancreatic or peripancreatic cystic disease, making preoperative diagnosis challenging.

**Patient concerns:**

We report a 43-year-old woman who presented with persistent dull left upper abdominal pain for 2 days. Laboratory tests showed nonspecific inflammatory and mild hepatobiliary abnormalities.

**Diagnoses:**

Ultrasonography and contrast-enhanced computed tomography demonstrated a giant multiloculated cystic lesion measuring approximately 160 × 110 mm and closely related to the pancreatic tail and splenic hilum. Histopathology demonstrated dilated endothelial-lined lymphatic spaces, and the written pathology report recorded D2-40 positivity, supporting the diagnosis of lymphangioma.

**Interventions:**

Exploratory laparotomy was performed. Because the lesion was densely adherent to the pancreatic tail and splenic hilum without a safe dissection plane, en bloc resection with distal pancreatectomy, splenectomy, and cholecystectomy was undertaken.

**Outcomes:**

Drain amylase was 1264 U/L on postoperative day 1 and 3337 U/L on postoperative day 3. The patient had no related symptoms, required no additional intervention, and had no delayed recovery, consistent with a biochemical leak under the 2016 International Study Group of Pancreatic Surgery definition. The drain was removed after ultrasonography showed no significant fluid collection, and the patient was discharged on postoperative day 11.

**Lessons:**

Lymphangioma should be considered in the differential diagnosis of giant multiloculated cystic lesions near the pancreatic tail and splenic hilum. When a symptomatic giant lesion cannot be safely separated from adjacent organs, complete en bloc resection may provide both definitive diagnosis and treatment.

## 1. Introduction

Lymphangioma is a benign lymphatic malformation composed of dilated lymphatic channels lined by endothelial cells and supported by connective tissue.^[1,2]^ Based on the size of the lymphatic spaces, lymphangiomas have traditionally been classified as capillary, cavernous, or cystic types.^[1,2]^ These lesions occur most often in children and typically arise in superficial locations, including the neck and axilla. Abdominal, retroperitoneal, pancreatic, and splenic-hilar involvement is uncommon.^[3–9]^ At this location, lymphangioma may resemble pancreatic pseudocyst, mucinous cystic neoplasm, intraductal papillary mucinous neoplasm (IPMN), mesenteric cyst, or other peritoneal cystic lesions, making preoperative diagnosis difficult. We report a rare case of a giant cystic lymphangioma in the pancreatic tail–splenic hilum region that required en bloc resection with distal pancreatectomy and splenectomy. The case highlights the diagnostic uncertainty of left upper abdominal cystic lesions, the intraoperative decision-making required when organ preservation is unsafe, and the importance of using current International Study Group of Pancreatic Surgery (ISGPS) terminology when interpreting postoperative drain-amylase elevation.

## 2. Case presentation

### 2.1. Patient information and clinical findings

A 43-year-old woman was admitted with persistent left upper abdominal pain for 2 days. The pain was dull, had no obvious trigger, and was accompanied by mild referred pain to the shoulder and back. She denied nausea, vomiting, jaundice, chills, fever, cessation of flatus or defecation, constipation, abdominal distension, or other discomfort. There were no clear aggravating or relieving factors. She had visited a local hospital and received symptomatic treatment with fluid infusion and analgesia, after which the pain partially improved. She then presented to our hospital for further evaluation and was admitted from the outpatient clinic with an initial working diagnosis of cholelithiasis with acute cholecystitis.

Her medical history was otherwise unremarkable. She denied hypertension, diabetes mellitus, and coronary heart disease. She had undergone remote gynecologic surgery more than 10 years earlier, but procedural details were unavailable. Personal and family histories were noncontributory.

On physical examination, vital signs were stable. The abdomen was distended, with tenderness in the left upper abdomen and questionable rebound tenderness. A relatively large smooth-surfaced mass was palpable in the left lower abdomen; this finding was considered compatible with inferior extension of the giant left-sided cystic lesion. No other remarkable findings were noted.

### 2.2. Diagnostic assessment

Laboratory tests after admission showed leukocytosis (white blood cell count 11.93 × 10^9^/L), elevated C-reactive protein (99.77 mg/L), and mildly elevated procalcitonin (0.100 ng/mL). Liver function tests revealed mildly elevated alanine aminotransferase (46 U/L), elevated total bilirubin (22.2 μmol/L), and decreased prealbumin (132 mg/L). Serum tumor markers, including CA19-9, carcinoembryonic antigen (CEA), and cancer antigen 125, were not obtained during the preoperative evaluation at our institution, and cyst-fluid CEA was not assessed. Therefore, pancreatic cystic neoplasms could not be completely excluded before surgery. Abdominal ultrasonography suggested a cystic mass in the left mid-upper abdomen. Contrast-enhanced upper abdominal computed tomography (CT) demonstrated a large multiloculated low-density cystic lesion with clear boundaries in the left abdominal cavity, measuring approximately 150 × 93 mm on the largest section. The lesion contained internal septations with mild septal enhancement and was indistinct from the pancreatic tail. The surrounding fat planes were slightly blurred, with scattered exudation/effusion and thickening of the adjacent peritoneum and mesentery (Fig. 1). CT also showed fatty liver, multiple gallbladder stones with possible cholecystitis, scattered splenic calcifications, and scattered retroperitoneal lymph nodes. Contrast-enhanced lower abdominal CT showed that the lesion measured approximately 160 × 110 mm on the largest section and suggested lymphangioma with surrounding exudation and effusion (Fig. 2). The working diagnoses were cholelithiasis with suspected acute cholecystitis and a giant cystic lesion suspicious for lymphangioma in the pancreatic tail–splenic hilum region. Magnetic resonance imaging (MRI) and endoscopic ultrasound-guided fine-needle aspiration (EUS-FNA) were not performed preoperatively because the lesion was symptomatic, giant, associated with inflammatory changes, and considered to have a surgical indication. CT had already demonstrated the size, extent, and relationship of the lesion to adjacent organs sufficiently for operative planning. EUS-FNA of a large multiloculated cystic lesion may also be limited by sampling error and would not necessarily have changed the need for surgical management in a symptomatic giant lesion. This incomplete preoperative cystic-lesion work-up is addressed as a limitation below.

**Figure 1.**
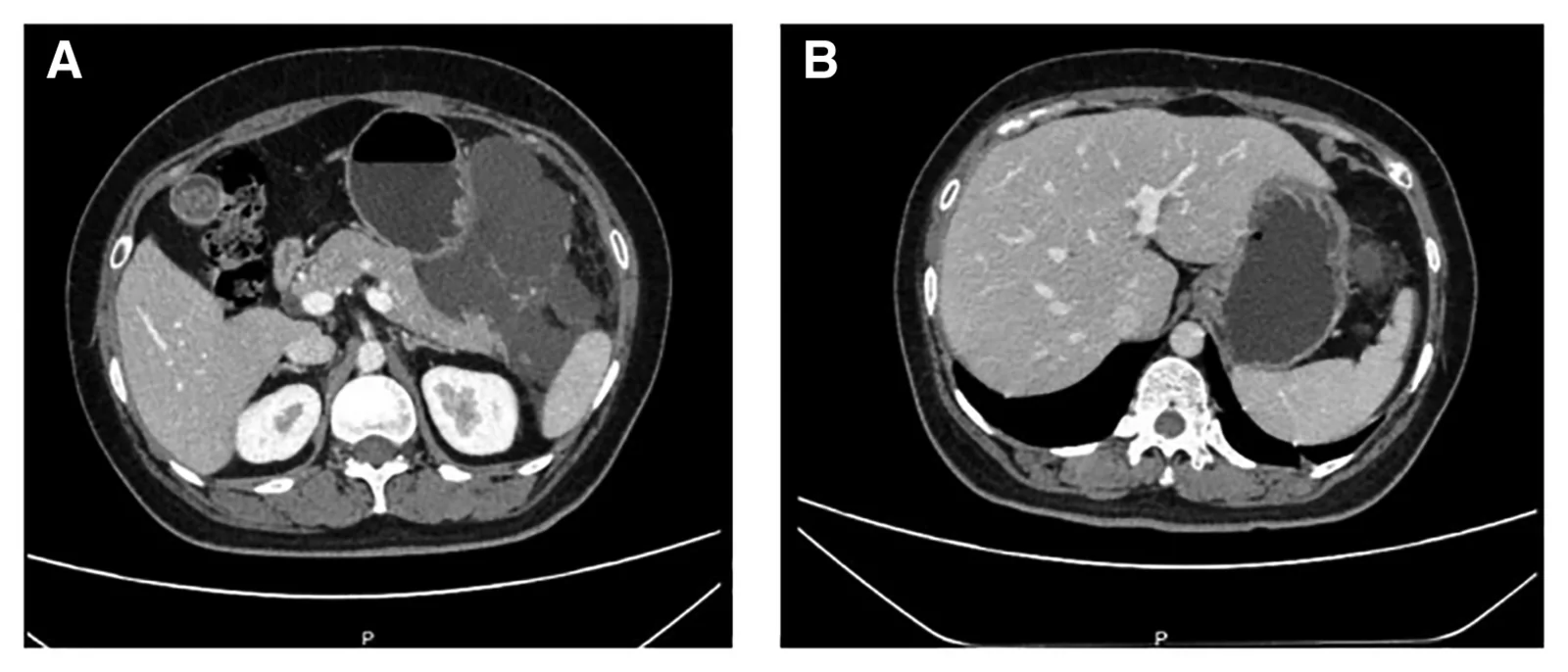
Contrast-enhanced upper abdominal CT. (A) Relationship between the mass, stomach, pancreas, and spleen. (B) Superior pole of the mass. CT showed a low-density, multiloculated lesion in the left abdominal cavity with septations and mild septal enhancement. The lesion was indistinct from the pancreatic tail and was associated with surrounding fat stranding, small effusions, and adjacent peritoneal/mesenteric thickening. CT = computed tomography.

**Figure 2.**
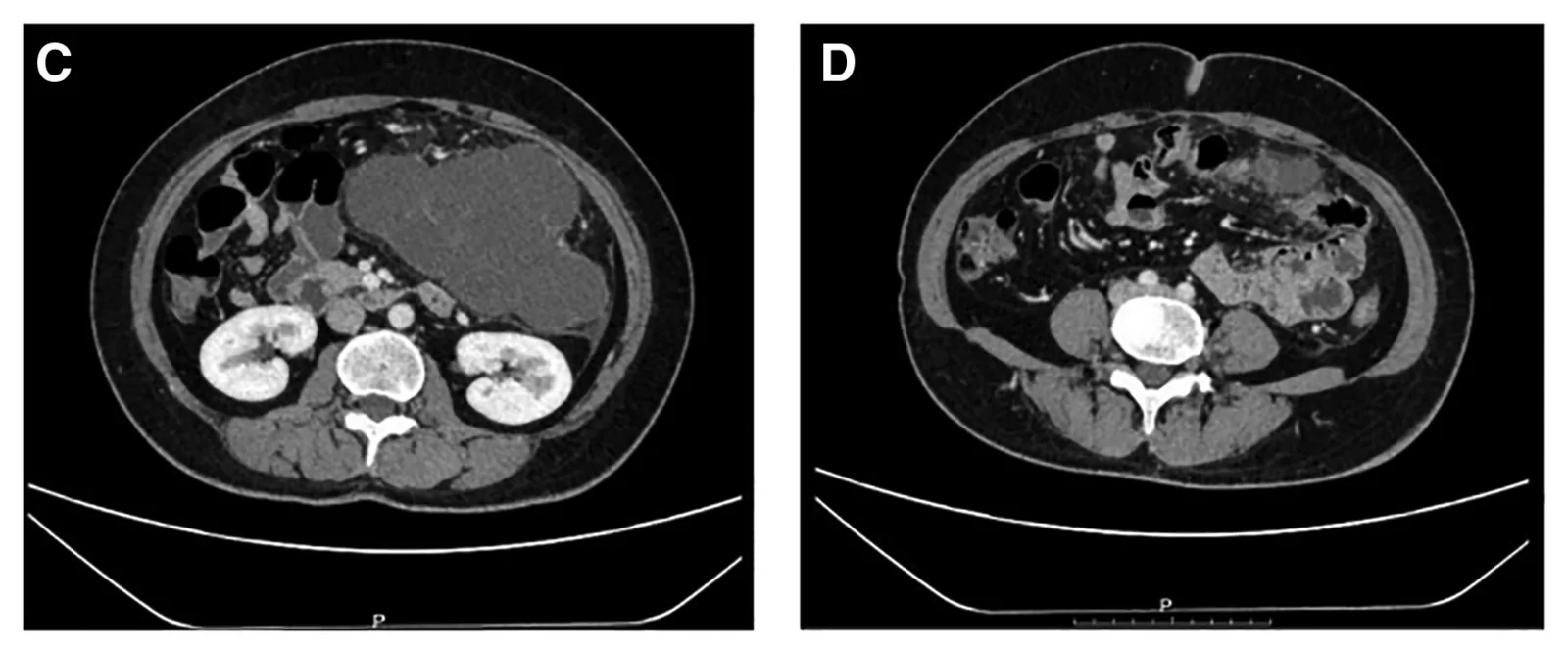
Contrast-enhanced lower abdominal CT. (C) Maximum cross-sectional plane of the mass. (D) Inferior pole of the mass. The lesion measured approximately 160 × 110 mm and was suspicious for lymphangioma with surrounding exudation and effusion. CT = computed tomography.

### 2.3. Timeline

The main diagnostic and therapeutic milestones are summarized in Table 1.

**Table 1:** Chronological clinical timeline.

Time point	Clinical event
Two days before admission	Onset of dull left upper abdominal pain with mild referred pain to the shoulder and back.
Admission	Initial working diagnosis of cholelithiasis with acute cholecystitis; laboratory tests showed inflammatory and mild hepatobiliary abnormalities.
Preoperative imaging	Ultrasonography and contrast-enhanced CT demonstrated a giant multiloculated cystic lesion closely related to the pancreatic tail and splenic hilum; lymphangioma was considered.
Operation	Exploratory laparotomy, en bloc resection of the lesion, distal pancreatectomy, splenectomy, and cholecystectomy were performed because no safe organ-preserving dissection plane was present.
POD 1 and POD 3	Drain amylase was 1264 U/L on POD 1 and 3337 U/L on POD 3; no related symptoms occurred and no additional intervention was required, consistent with a biochemical leak.
POD 4	Postoperative contrast-enhanced CT showed scattered exudation/effusion around the operative field with drainage tubes in situ.
POD 7	Follow-up CT showed slightly decreased exudation/effusion.
POD 9	Ultrasonography showed no significant fluid accumulation; the drainage tube was subsequently removed.
POD 11	The patient improved and was discharged.

POD = postoperative day.

### 2.4. Therapeutic intervention

Three operative strategies were discussed preoperatively: lesion resection alone, lesion resection with distal pancreatic resection, and lesion resection with distal pancreatic resection plus splenectomy. The preoperative informed discussion included the possibility of distal pancreatectomy and/or splenectomy if complete and safe lesion removal could not be achieved by lesion excision alone. Exploratory laparotomy with lesion resection and cholecystectomy was initially planned, with possible distal pancreatectomy and/or splenectomy depending on intraoperative findings.

Intraoperatively, a huge, multiloculated, dark red-black mass was identified in the abdominal cavity. The lesion had obvious adhesions to the stomach and greater omentum and dense adhesions to the pancreatic tail and splenic hilum, without a clear dissection plane (Fig. 3). Cyst fluid was aspirated intraoperatively for amylase testing. The cyst-fluid amylase level was 85 U/L, well below the 250 U/L threshold reported to virtually exclude a pancreatic pseudocyst, arguing against a pancreatic pseudocyst or communication with the pancreatic duct.^[10]^ During mobilization, mild adhesions to the stomach, greater omentum, and descending colon were released using blunt and sharp dissection. Because the lesion was closely related to the pancreatic tail and splenic hilum and could not be separated safely, an organ-preserving approach was not feasible. Intraoperative frozen section was not used to guide organ preservation because sampling of a large multiloculated cystic lesion may be limited by sampling error and would not reliably exclude focal cystic neoplasm across the entire lesion. After repeated intraoperative communication with the patient’s family regarding the pre-discussed extended operative strategy, en bloc resection of the lesion with distal pancreatectomy, splenectomy, and cholecystectomy was performed to achieve complete removal and avoid residual disease.

**Figure 3.**
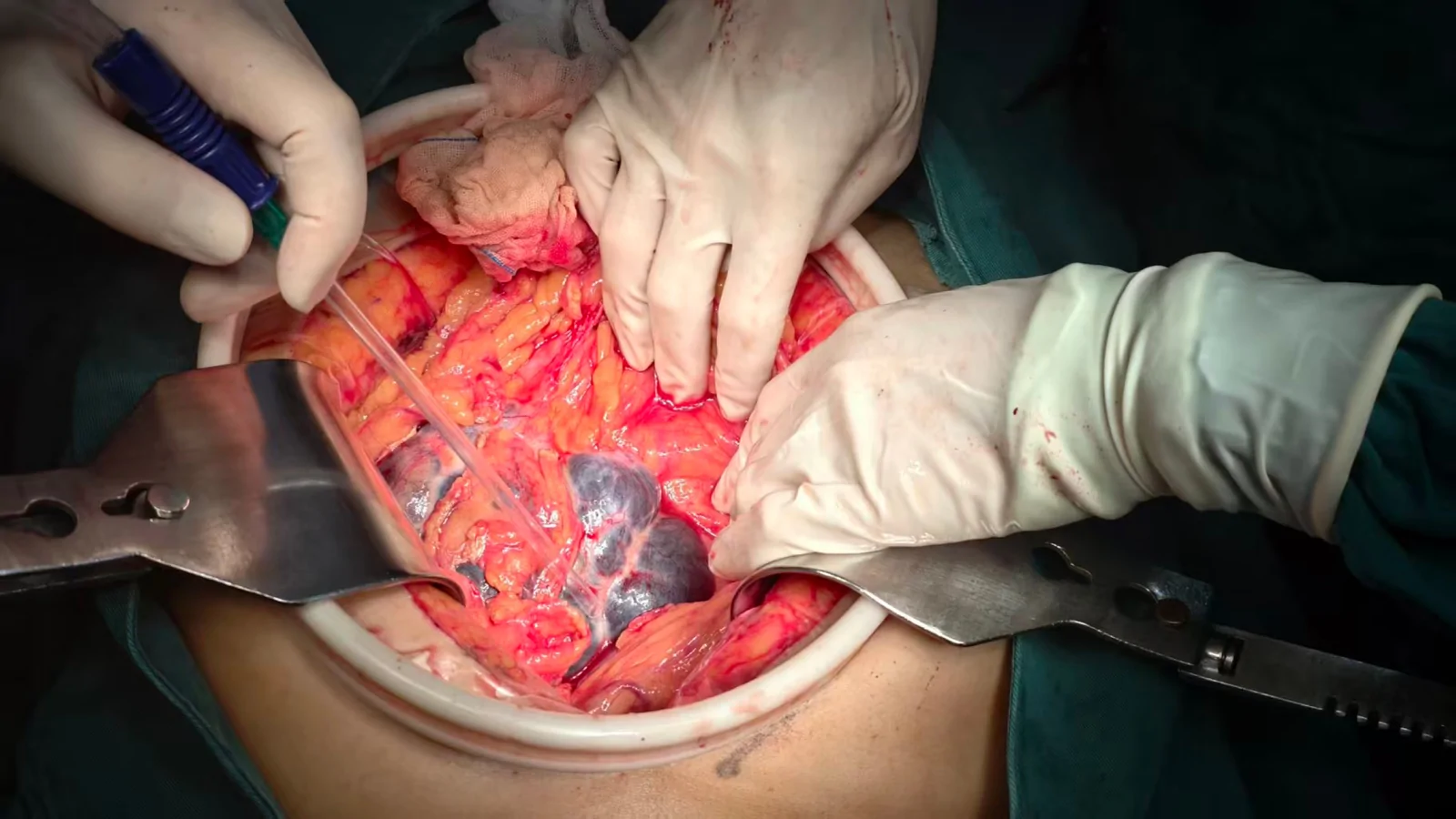
Intraoperative findings. A huge, multiloculated, dark red–black mass was densely adherent to the stomach, greater omentum, pancreatic tail, and splenic hilum.

### 2.5. Histopathological findings and final diagnosis

Postoperative histopathology showed dilated endothelial-lined lymphatic spaces. The written pathology report recorded D2-40 positivity, supporting lymphatic endothelial differentiation (Fig. 4). The resected specimen included the lesion, distal pancreas, and spleen. The final diagnosis was lymphangioma in the pancreatic tail–splenic hilum region. Because no immune histochemical photograph was available for publication, only the hematoxylin-eosin image is presented.

**Figure 4.**
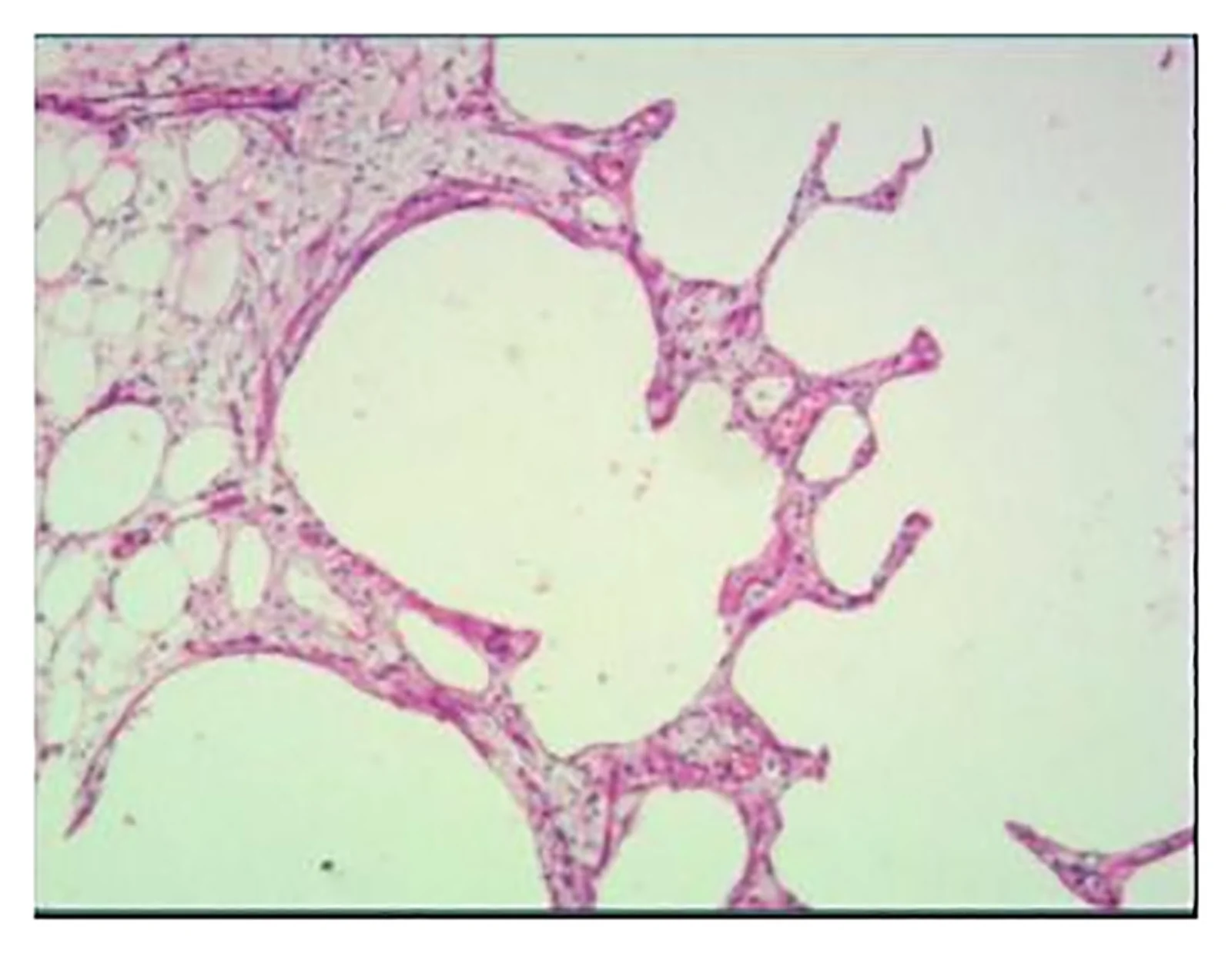
Histopathology. Hematoxylin-eosin staining showed dilated, endothelial-lined lymphatic spaces, consistent with lymphangioma. The written pathology report recorded D2-40 positivity, supporting lymphatic endothelial differentiation; however, no D2-40 immunohistochemical photograph was available for publication.

### 2.6. Follow-up and outcomes

Drain amylase was 1264 U/L on postoperative day 1(POD) and increased to 3337 U/L on POD 3 (institutional serum amylase reference range, 35–135 U/L), exceeding 3 times the institutional upper limit of normal serum amylase on POD 3. The patient had no symptoms, required no additional intervention, and had no delayed recovery. According to the 2016 ISGPS update, this finding is best classified as a biochemical leak rather than a clinically relevant postoperative pancreatic fistula (POPF) because it had no clinical impact.

Postoperative contrast-enhanced CT on POD 4 showed scattered exudation and effusion in and around the operative area, local swelling of the greater omentum, and drainage tubes in situ in the left upper abdomen and gallbladder fossa (Fig. 5). Follow-up CT on POD 7 demonstrated slightly decreased exudation/effusion compared with prior imaging. On POD 9, abdominal ultrasonography revealed no significant fluid accumulation, and the drainage tube was subsequently removed. The patient improved and was discharged on POD 11.

**Figure 5.**
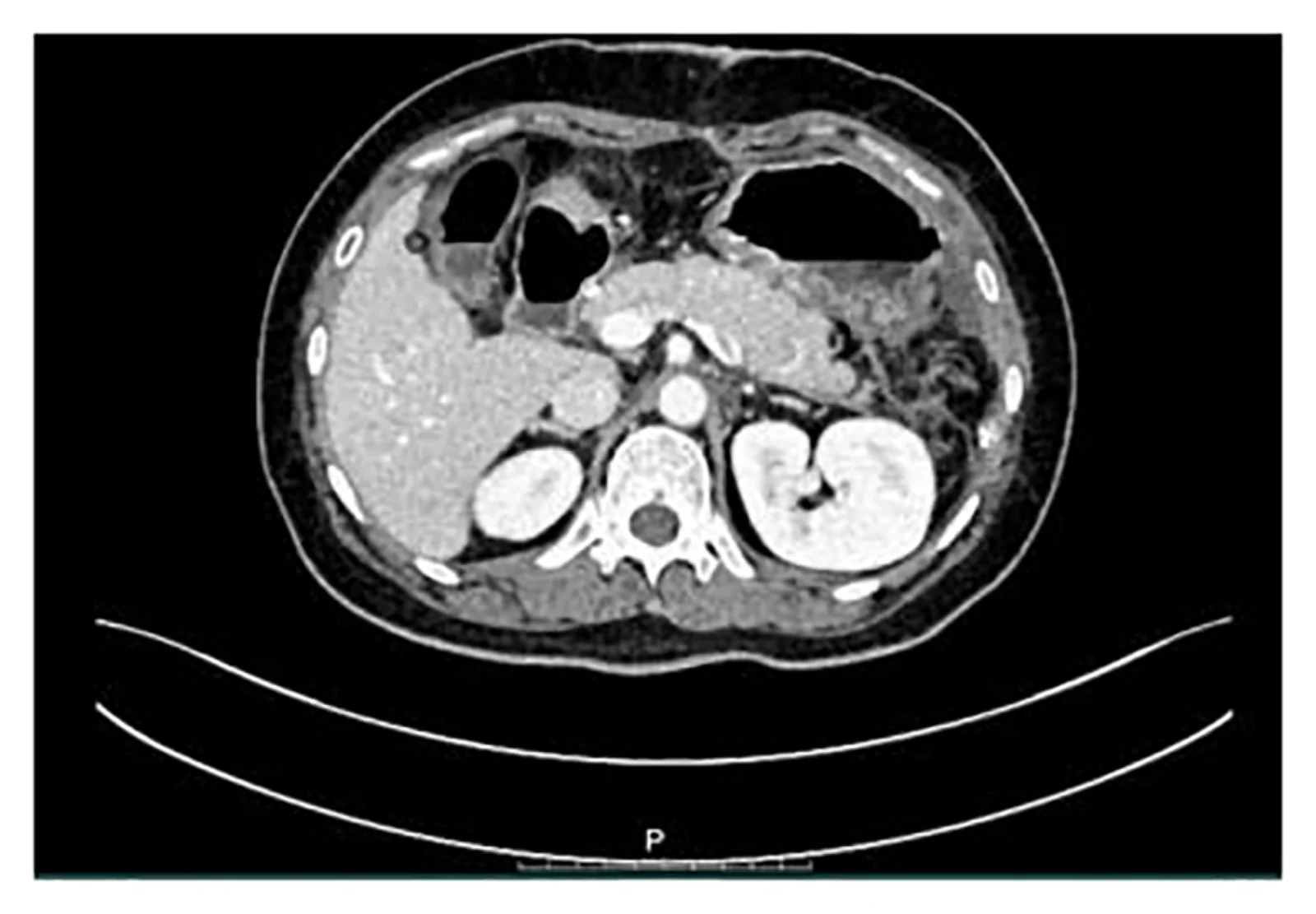
Postoperative contrast-enhanced CT. Scattered exudation/effusion was seen around the operative site with local swelling of the greater omentum and drainage tubes in situ. CT = computed tomography.

## 3. Discussion

Lymphangioma is a benign lesion thought to result from congenital lymphatic developmental abnormalities or impaired lymphatic drainage; however, its etiology remains unclear.^[1,3,4]^ Most investigators consider it a congenital malformation in which aberrant lymphatic channels fail to drain properly, leading to abnormal lymphatic dilation and proliferation.^[1,3,4]^ Other proposed mechanisms include trauma, inflammation, and long-term lymphatic obstruction.^[1,11]^ Although benign, abdominal lymphangioma can grow along tissue planes and may compress or adhere to adjacent organs.^[5,12–15]^

This case illustrates why lymphangioma in the pancreatic tail–splenic hilum region is diagnostically difficult. Clinical manifestations are nonspecific and may be absent until the lesion becomes large or is complicated by hemorrhage, infection, inflammation, or compression.^[1,3–5,11–13,16]^ In this patient, leukocytosis and elevated C-reactive protein were nonspecific inflammatory findings that may have been related to perilesional inflammatory exudation on CT and/or concomitant cholelithiasis with suspected cholecystitis. These abnormalities did not indicate a specific diagnosis but were consistent with the symptomatic presentation.

Imaging plays a central role in the evaluation of abdominal lymphangioma. Ultrasonography usually shows a well-demarcated cystic or multiloculated mass with internal septa and minimal internal blood flow. CT commonly demonstrates a thin-walled, low-density cystic lesion, often with multilocular septations and mild enhancement of the cyst wall or septa. When infection, hemorrhage, or proteinaceous content is present, wall thickening, surrounding fat stranding, increased attenuation, or fluid-fluid levels may be observed. MRI can better demonstrate septa and cyst contents and is useful for differentiating lymphangioma from pancreatic cystic neoplasms. In a recent series of 28 adult retroperitoneal lymphangiomas, CT was performed in all patients, whereas MRI was obtained in only 4 patients (14.3%), illustrating that MRI was not universally used in that clinical series.^[6,11,14,16]^

The main differential diagnoses in this case included pancreatic pseudocyst or walled-off fluid collection, pancreatic cystic neoplasms such as mucinous cystic neoplasm, IPMN, or cystic degeneration of solid pseudopapillary neoplasm, mesenteric or omental cysts, retroperitoneal cystic lesions, cystic mesothelioma, cystic metastases, and pseudomyxoma peritonei.^[2,7–9,11,16,17]^ The low intraoperative cyst-fluid amylase level (85 U/L), well below the 250 U/L threshold reported to virtually exclude a pancreatic pseudocyst, and the absence of a documented pancreatitis history argued against a pancreatic pseudocyst or pancreatic duct communication.^[10]^ No definite mural nodule, papillary projection, or pancreatic duct communication was described on CT. However, the absence of serum tumor marker data, cyst-fluid CEA, preoperative MRI, and EUS-FNA limited preoperative discrimination from pancreatic cystic neoplasms, including mucinous cystic neoplasm, IPMN, and cystic degeneration of solid pseudopapillary neoplasm. Definitive diagnosis therefore depended on histopathology.

Histologically, cystic lymphangioma typically consists of dilated lymphatic spaces lined by a single layer of endothelial cells. D2–40 is a marker of lymphatic endothelium. In the present case, the pathological morphology together with D2–40 positivity recorded in the written pathology report supported the final diagnosis.^[2,7,8]^

Complete surgical excision is generally preferred for symptomatic or large lymphangiomas because it provides a definitive diagnosis, relieves symptoms, and reduces recurrence risk.^[3,11,13–15]^ Less invasive options, including aspiration, sclerotherapy, or subtotal resection with chemical cauterization, may be considered in selected cases when complete excision is unsafe.^[11,15]^ In this case, the lesion was densely adherent to the pancreatic tail and splenic hilum, and no safe plane existed for spleen- or pancreas-preserving resection. En bloc distal pancreatectomy and splenectomy were therefore selected to achieve complete resection. Frozen section has potential value in selected intraoperative decisions, but in a giant multiloculated cystic lesion, sampling error may limit its ability to exclude focal neoplastic elements. Moreover, in this case, the decisive factor was the absence of a safe dissection plane rather than the histologic diagnosis alone.

The postoperative drain-amylase elevation in this case should be interpreted using updated terminology. The 2016 ISGPS update reclassified the former grade A POPF as a biochemical leak, which has no clinical importance and is not considered a true pancreatic fistula.^[18]^ In this patient, drain amylase was 3337 U/L on POD 3, exceeding 3 times the institutional upper limit of normal serum amylase (135 U/L), but there were no symptoms, additional interventions, or delayed recovery. The drain was removed after imaging confirmed no significant fluid accumulation, and the patient was discharged on POD 11.

### 3.1. Limitations

This case has several limitations. First, serum tumor markers, including CA19-9, CEA, and cancer antigen 125, were not obtained during the preoperative evaluation at our institution, and cyst-fluid CEA was not assessed. Second, preoperative MRI and EUS-FNA were not performed, which limited preoperative discrimination from pancreatic cystic neoplasms. Third, D2–40 positivity was recorded in the written pathology report, but no immunohistochemical photograph was available for publication. Fourth, documentation regarding postsplenectomy vaccination and infection-risk counseling was unavailable. Patients undergoing splenectomy for benign disease should receive appropriate vaccination and infection-risk counseling according to local guidelines. Finally, follow-up was limited to the early postoperative period; longer-term imaging follow-up remains necessary to evaluate late recurrence.

## 4. Conclusion

Lymphangioma in the pancreatic tail–splenic hilum region is rare and may present with nonspecific symptoms and nonspecific laboratory abnormalities. It should be included in the differential diagnosis of giant multiloculated cystic lesions of the left upper abdomen. Complete en bloc resection can be both diagnostic and therapeutic when the lesion is symptomatic, large, closely related to the pancreatic tail and splenic hilum, and cannot be safely separated from adjacent organs. In this case, the postoperative course was favorable during hospitalization; however, longer follow-up is still needed to evaluate late recurrence. Transient drain-amylase elevation after distal pancreatectomy should be classified according to the 2016 ISGPS update and, when clinically silent, described as a biochemical leak rather than a true POPF.^[18]^

## Author contributions

**Writing – original draft:** Yuting Wang.

**Writing – review & editing:** Bin Xiong, Junwei Niu, Shengwei Li.
